# Idiopathic Intestinal Smooth Muscle Hyperplasia in a French Bulldog: Clinical, Imaging, Capsule Endoscopy, and Histopathological Findings

**DOI:** 10.3390/ani15091199

**Published:** 2025-04-23

**Authors:** Hyomi Jang, Sang-Woo Kim, Joon Woo Lee, Munso Kim, Dong-In Jung

**Affiliations:** 1 VIP Animal Medical Center (Cheongdam), Seoul 06068, Republic of Korea; hm100su@gmail.com; 2 College of Veterinary Medicine, Gyeongsang National University, Jinju 52828, Republic of Korea; camsang90@naver.com (S.-W.K.); joon9108@kakao.com (J.W.L.); kimmunso0801@gmail.com (M.K.)

**Keywords:** anastomosis, capsule endoscopy, dog, hyperplasia

## Abstract

A 3-year-old intact female French Bulldog presented with chronic diarrhea, intermittent vomiting, hyporexia, and weight loss over seven weeks. The blood test results were unremarkable, except for mild hypocholesterolemia. Abdominal ultrasonography revealed intestinal wall thickening, particularly in the muscular layer, with focal loss of wall layering in the distal ileum near the ileocecal junction. Capsule endoscopy revealed mild-to-moderate mucosal irregularities and changes in the ileum, with a reduced luminal diameter caused by irregularly protruding walls, leading to the partial obstruction of the distal ileum. To remove the obstructed segment, surgical resection and anastomosis were performed from the distal ileum to the ileocecal junction, including the ileocolic valve region. Histopathological examination confirmed intestinal smooth muscle hyperplasia with mild infiltration of macrophages, neutrophils, and lymphocytes. As the underlying cause could not be identified, the condition was classified as an idiopathic condition. Postoperatively, long-term management of the clinical symptoms was implemented, including antibiotics, anti-inflammatory medications, and dietary modifications (the elimination of table foods). Over a one-year follow-up period, ultrasonography revealed mild recurrent inflammation and muscular hyperplasia; however, the clinical signs resolved, except for occasional episodes of diarrhea.

## Simple Summary

A 3-year-old French Bulldog was evaluated for chronic diarrhea, vomiting, and weight loss. Diagnostic imaging, including ultrasonography and capsule endoscopy, revealed thickening of the intestinal muscular layer and partial ileal obstruction. Surgical removal of the affected intestinal segment confirmed the rare condition of intestinal smooth muscle hyperplasia. No specific underlying cause was identified, and the condition was classified as idiopathic. The dog was administered long-term anti-inflammatory medication, antibiotics, and dietary supplements. Over one year of follow-up, mild intestinal thickening persisted; however, the clinical signs improved significantly, with only occasional diarrhea. This case highlights the importance of advanced diagnostic and surgical interventions for rare intestinal disorders in dogs.

## 1. Introduction

Idiopathic smooth muscle hyperplasia is a rare condition in veterinary medicine, characterized by the non-neoplastic proliferation of smooth muscle layers within the gastrointestinal tract [1]. Unlike inflammatory conditions such as inflammatory bowel disease (IBD) or chronic intestinal pseudo-obstruction (CIPO), idiopathic smooth muscle hyperplasia primarily involves the thickening of the *tunica muscularis* without significant inflammatory infiltration [2,3,4,5]. Few reports have documented this condition in animals, and its pathogenesis remains largely unknown [1,6,7,8]. Similar conditions have been described in humans, but their clinical significance is not well established [9,10,11,12,13].

In humans, a few cases of gastrointestinal muscular hypertrophy of unknown etiology, primarily affecting the *muscularis propria*, have been documented [9,10,11,12,13]. This condition is rarely observed and is typically asymptotic.

Smooth muscle hypertrophy of the gastrointestinal tract has been reported in several species, including horses, rabbits, and cats [6,7,8]. However, clinical presentation, diagnostic approaches, and treatment strategies vary significantly among species. In horses, idiopathic muscular hypertrophy of the small intestine is frequently associated with colic and can result in intestinal obstruction, requiring surgical intervention [6]. Similarly, in cats, the hypertrophy of the intestinal *tunica muscularis* has been linked to chronic enteropathy and idiopathic inflammatory processes [7]. The mechanisms underlying this condition remain speculative, with potential contributions from chronic motility disorders, genetic predisposition, and subclinical inflammation [6,7,8].

This report describes a case of idiopathic intestinal smooth muscle hyperplasia in a young dog, presenting with gastrointestinal symptoms. The diagnosis was confirmed using imaging, capsule endoscopy, surgical resection, and histopathological evaluation.

## 2. Case Description

A 3-year-old intact female French Bulldog weighing 10.1 kg presented with chronic diarrhea, intermittent vomiting, and hyporexia for seven weeks. The dog lost approximately 1 kg of body weight after the onset of the clinical signs. According to history, a dietary change to a low-fat gastrointestinal i/d diet (Hill’s Pet Nutrition, Topeka, KS, USA) was previously initiated by a local veterinary clinic due to chronic diarrhea. Stool quality showed temporary improvement during the dietary change. However, diarrhea recurred after the reintroduction of beef, which was fed due to the dog’s poor appetite and refusal to continue eating commercial diets. The patient did not receive preventive care with vaccines or antiparasitic drugs. According to the owner, the dog lived exclusively indoors with minimal outdoor activity and had no regular contact with other animals. As a result, the owner believed that the dog was at low risk of infection and did not perceive the need for routine preventive care. In addition, no medical interventions other than dietary changes were attempted before visiting the referral hospital.

Physical examination revealed abdominal distension. The blood samples were collected after a fasting period of more than 12 h. Mild hypocholesterolemia (106 mg/dL; reference interval: 110–320 mg/dL, Catalyst One Chemistry Analyzer, IDEXX Laboratories, Westbrook, ME, USA) and hyperchloremia (114.8 mg/dL; reference interval: 107–113 mg/dL, pHOx Ultra Blood Gas Analyzer, Nova Biomedical, Waltham, MA, USA) were noted, with no significant abnormalities in the complete blood count.

**Figure 1:**
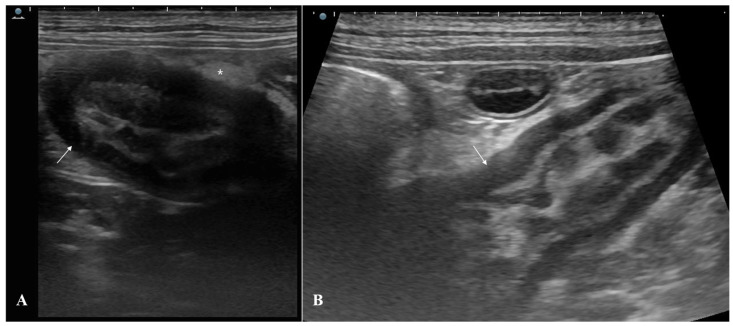
Abdominal ultrasonography of the ileum revealed thickened walls. Diffuse wall thickening and muscular layer hypertrophy ((**A**,**B**): arrows) were observed in the distal ileum adjacent to the ileocecal junction. Hyperechoic changes ((**A**): asterisk) in the surrounding mesenteric fat were also observed.

Abdominal radiography revealed no significant abnormalities. Abdominal ultrasonography revealed diffuse wall thickening (8.5 mm; reference: <2.5 mm) and muscular layer thickening (4.7 mm; reference: <2.0 mm) in the distal ileum near the ileocecal junction [9] (Figure 1). Loss of focal wall layering and mild corrugation were also noted, along with hyperechoic changes in the surrounding mesenteric fat. These findings suggest the possibility of intestinal neoplasia originating in the muscular layer. However, severe intestinal inflammation cannot be excluded.

**Figure 2:**
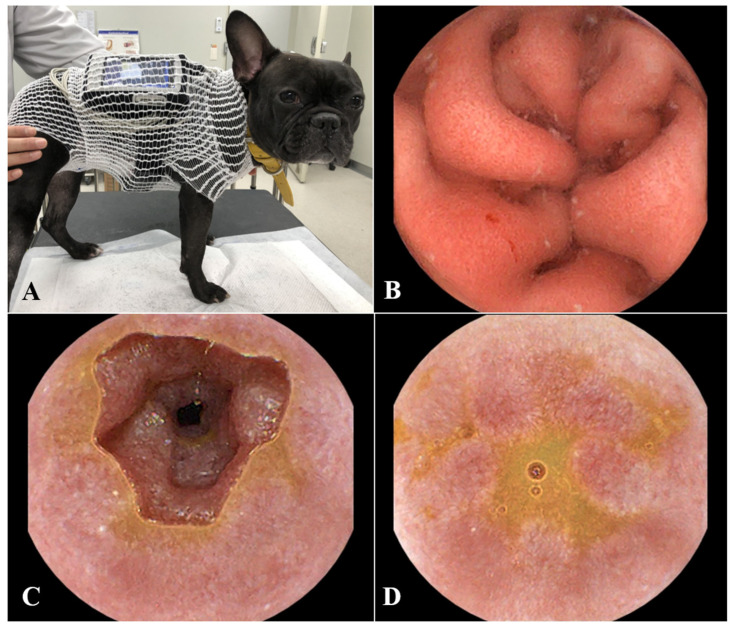
The patient was fitted with capsule endoscopy equipment (**A**). Capsule endoscopy of the stomach (**B**) revealed a bleeding spot and mild erythema of the gastric mucosa. Images of the ileum (**C**,**D**) showed moderate mucosal irregularities, erythematous changes, and an intraluminal protrusion causing partial obstruction of the distal ileum.

Wireless capsule endoscopy was performed using the MiroCam^®^ (MC-1200M, Intromedic, Seoul, Republic of Korea) to assess mucosal changes and check for intestinal obstruction (Figure 2A). A few bleeding spots and mild erythema were observed in the gastric mucosa (Figure 2B); however, no abnormal lesions were observed in the duodenum or jejunum. Nevertheless, mild-to-moderate mucosal irregularities were observed in the ileum, where the luminal diameter was reduced due to irregularly protruding walls, leading to a partial obstruction of the distal ileum (Figure 2C,D).

The differential diagnoses included inflammatory bowel disease, lipogranulomatous lymphangitis, leiomyositis, intestinal smooth muscle neoplasia and granulomatous enteropathy.

**Figure 3:**
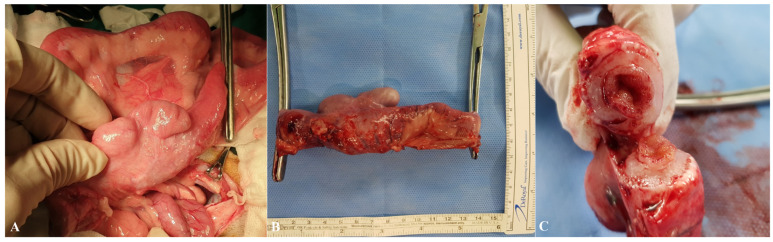
Gross observation of partially enlarged ileal segments near the ileocolic valve (**A**). Thirteen centimeters of the distal ileum were surgically resected (**B**). The cross-section of the resected ileum (**C**) revealed severe wall thickening and narrowing of the intraluminal space.

Therefore, we opted for a surgical approach for histopathological confirmation. Surgical resection and anastomosis were performed from the distal ileum to the ileocecal junction by removing the obstructed segment, including the ileocolic valve region (Figure 3). Approximately 13 cm of the distal ileum was resected. A full-thickness biopsy sample, including the hypertrophic muscle layer, was collected and five representative cross-sectional tissue blocks from the resected segment were submitted for histopathological analysis.

**Figure 4:**
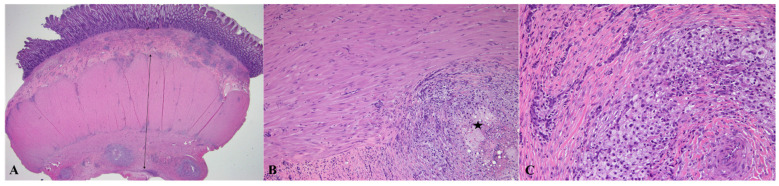
Histopathological examination confirmed severe thickening of the *muscularis propria* ((**A**): H&E, ×12.5, arrow) with mild-to-moderate infiltration of macrophages, neutrophils, and lymphocytes ((**B**): H&E, ×100, asterisk, (**C**): H&E, ×200).

Histopathological examination revealed the significant thickening of the intestinal muscular layer, but no invasion of the mucosa or submucosa by the muscle. At high magnification, the proliferating smooth muscle cells exhibited irregular and random orientations, which differed from the original alignment. Although no evidence of inflammatory cell infiltration was observed in the intestinal mucosa, the mild-to-moderate multifocal infiltration of inflammatory cells, including macrophages, neutrophils, and lymphocytes, was observed in the proliferating *tunica muscularis* layer. In the submucosa, a similar degree of inflammatory infiltration was also identified. No infectious agents, such as bacteria or fungi, were detected on routine histopathological evaluation. The histopathological findings are shown in Figure 4. No evidence of neoplastic cells was observed in any of the samples.

Based on the medical history and histopathological findings, the patient was diagnosed with idiopathic intestinal smooth muscle hyperplasia with mild infiltration of macrophages, neutrophils, and lymphocytes. As the underlying cause could not be identified, the condition was classified as idiopathic.

Based on the histopathological findings of mild-to-moderate infiltration of inflammatory cells in the submucosa and *tunica muscularis*, anti-inflammatory treatment with prednisolone (1 mg/kg, PO, q24h, tapered for a few weeks) was initiated. Tylosin (25 mg/kg, PO, q24h) was concurrently prescribed to minimize intestinal dysbiosis and support mucosal recovery. Strict elimination of table foods was also implemented to prevent further gastrointestinal irritation. Six weeks after surgery, ultrasonography revealed mild mucosal inflammation in the small intestine. During the one-year follow-up period, mild muscular thickening persisted; however, the clinical signs resolved, except for occasional diarrhea. The patient’s body weight increased to 11.8 kg. During the follow-up period, no abnormalities were detected in the blood tests.

## 3. Discussion

This report describes a rare case of idiopathic intestinal smooth muscle hyperplasia in a dog, characterized by chronic gastrointestinal symptoms, including chronic diarrhea, intermittent vomiting, and weight loss. Idiopathic smooth muscle hyperplasia in dogs is rarely reported and is often mistaken for neoplastic processes, such as leiomyoma or gastrointestinal stromal tumors. In the present case, the absence of neoplastic cellular atypia and significant inflammatory infiltration distinguished this condition from other inflammatory and neoplastic disorders of the intestine.

Sharp et al. [1] reported a similar case of smooth muscle hyperplasia in a dog, in which a solitary mass at the ileocolic junction was found to be non-neoplastic upon histopathological examination. Unlike this case, which involved an older dog, our patient was young and exhibited diffuse muscular thickening from the distal ileum to the ileocecal junction. These findings suggest that idiopathic smooth muscle hyperplasia may present in different forms depending on the affected region and extent of involvement.

The pathogenesis of idiopathic smooth muscle hyperplasia remains unknown [1,2]. Various hypotheses have been proposed, including the chronic low-grade stimulation of smooth muscle proliferation due to altered motility, subclinical inflammation, and genetic predisposition [2]. Bettini et al. [7] suggested a potential link between intestinal inflammation and smooth muscle hypertrophy in cats, suggesting that inflammatory mediators act as hypertrophic stimuli in certain cases. However, in the present case, no significant inflammatory response was observed, supporting the notion that idiopathic smooth muscle hyperplasia represents a distinct pathological entity rather than an inflammatory condition. It is highly likely that mild inflammation in this case was a secondary change, rather than a primary pathological process. A recent case report of a similar condition in a Bulldog documented only mild inflammation [1].

Reports of similar conditions in other species underscore the complexities of this disorder. In equines, idiopathic muscular hypertrophy of the small intestine is associated with chronic colic and functional obstruction [6]. In rabbits, smooth muscle hypertrophy is linked to pseudodiverticulosis, which can lead to peritonitis and severe gastrointestinal dysfunction [8]. These variations suggest that species-specific factors may influence the clinical manifestations and progression of smooth muscle hyperplasia [8].

Few previous studies in humans have documented gastrointestinal muscular hypertrophy of unknown etiology, predominantly affecting the *muscularis propria* with limited inflammatory infiltration [10,11,12,13,14]. Muscular hypertrophy, fibrosis, and fibromuscular proliferation are prominent changes in Crohn’s disease (CD), a form of inflammatory bowel disease (IBD) in humans [15]. Interestingly, in humans, CD involves muscular hypertrophy; however, these changes are typically accompanied by significant inflammation in the mucosal and submucosal layers, which was absent in this canine case [15,16]. Canine IBD is another well-known condition that can cause thickening of the intestinal wall; however, its hallmark histopathological feature is the presence of infiltrative inflammatory cells in the mucosa and submucosa, similar to CD [5]. Therefore, the absence of mucosal inflammation further distinguishes this case from CD in humans and IBD in dogs.

The use of advanced diagnostic modalities, such as ultrasonography, capsule endoscopy, and surgical biopsy, was crucial in this case. Capsule endoscopy allowed a detailed visualization of the mucosal surfaces and revealed partial obstruction caused by protruding walls in the distal ileum. Additionally, capsule endoscopy allows for a direct visual assessment of the mucosal condition and the presence or absence of lacteal dilation. Although increasingly employed in human gastroenterology, this technology has limited applications in veterinary medicine and has proven valuable for assessing mucosal changes beyond the reach of standard endoscopy [17,18,19,20,21,22,23,24,25]. A key advantage of capsule endoscopy is that it does not require sedation or anesthesia. However, in cases of significant functional or mechanical obstruction, the device may become trapped in the intestine, potentially causing serious complications such as iatrogenic foreign body formation or perforation secondary to device retention [26]. In human medicine, computed tomography, magnetic resonance imaging, and the use of potent dissolvable capsules that break down in the intestine after several hours have been used to reduce these risks [27]. However, owing to concerns regarding anesthesia and cost, a preliminary gastrointestinal ultrasound was performed to check for severe *ileus* or complete obstruction before capsule endoscopy. These findings confirmed that the partially obstructed lesions had sufficient diameter for the device to pass through. The effectiveness of preliminary ultrasounds in preventing complications has also been reported in human medicine [28]. A definitive diagnosis was made through a full-thickness biopsy performed during surgical intervention, as the primary lesion was located in the *muscularis propria*. The differential diagnoses included gastrointestinal neoplasms such as leiomyoma, leiomyosarcoma, gastrointestinal stromal tumor (GIST), lymphoma, and adenocarcinoma. However, the absence of neoplastic features—including cellular atypia, mitotic figures, and evidence of tissue invasion—combined with the favorable clinical response, supported a diagnosis of non-neoplastic smooth muscle hyperplasia.

A limitation of this case is that immunohistochemical (IHC) staining was not performed. Although the histopathological findings—including the absence of atypia and mitotic figures—supported a diagnosis of non-neoplastic smooth muscle hyperplasia, IHC staining could have further strengthened the diagnostic certainty by excluding neoplastic differentials such as GISTs.

Several ancillary diagnostic procedures were also not performed, which represents an additional limitation. Antiparasitic treatment was not administered, as there was no clinical evidence suggesting parasitic infection. Advanced infectious disease testing, including fecal PCR or molecular assays, was not pursued due to financial constraints expressed by the owner. The assessment of gastrointestinal absorption parameters, such as serum vitamin B12, folate, and trypsin-like immunoreactivity (TLI), was not conducted but is acknowledged as a valuable diagnostic approach in chronic enteropathies. In this case, surgical biopsy was prioritized over less invasive sampling due to the obstructive nature of the lesion, necessitating immediate resection. The entire resected segment was examined macroscopically, and five representative full-thickness samples were evaluated histologically. Although postoperative malabsorption was considered a potential complication, the patient’s appetite, fecal consistency, and body weight improved following surgery, supporting an overall positive outcome.

The appropriate treatment for idiopathic intestinal smooth muscle hyperplasia in dogs has not been established. In this case, the therapeutic approach combined surgical intervention with dietary changes and anti-inflammatory and antibiotic therapies, resulting in the resolution of clinical signs and weight gain during the follow-up period. The persistence of mild muscle thickening despite treatment suggests a chronic, possibly irreversible condition. However, the resolution of clinical signs, with only occasional diarrhea, indicated a favorable response to the chosen management strategy. This outcome underscores the importance of surgical resection in cases where conservative management fails to alleviate symptoms and highlights the potential benefits of anti-inflammatory therapy with antibiotics and dietary changes.

## 4. Conclusions

This case emphasizes the importance of considering idiopathic smooth muscle hyperplasia in the differential diagnosis of dogs with chronic gastrointestinal symptoms and muscle hypertrophy. Further studies are required to elucidate the underlying pathophysiology and optimal management strategies for this rare condition.

## Data Availability

The original contributions of this study are presented in this article. Further inquiries can be directed to the corresponding authors.
